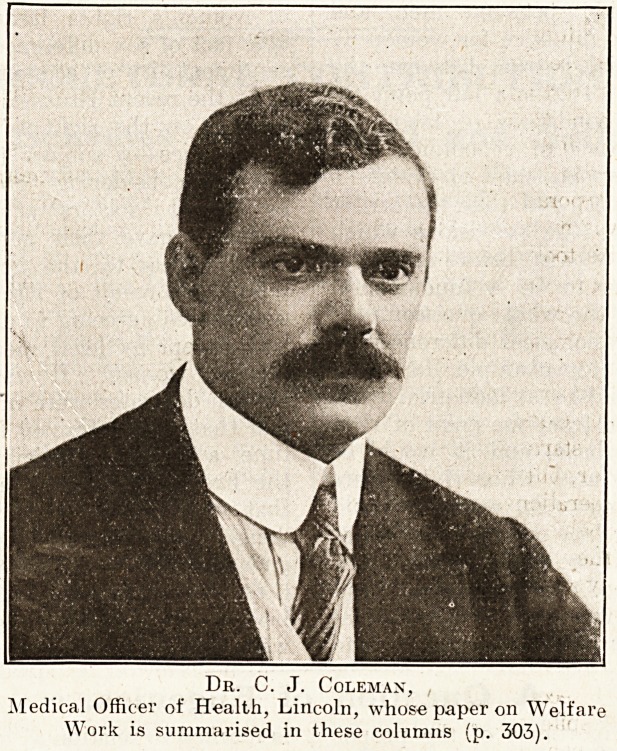# Medical War Service

**Published:** 1920-06-19

**Authors:** 


### Medical War Service.

### A Great Record and a Great Tribute.

The remarkable achievements of the Royal Army Medical Department during the war received renewed recognition last week at a dinner in honour of the late Director-General of Medical Services, Sir Alfred Keogh. There was a distinguished gathering, including a former War Secretary, the Earl of Midleton, who presided, the present War Secretary (Mr. Churchill), Earl Haig, the Earl of Derby, and many others. The chairman said no more than the truth in declaring that if a review were to be held of the departments of the Army, and if the award of excellence were given solely for progress since 1900, the Army Medical Department might very likely be placed on the right of the line.

Dr. C. J. Coleman,
Medical Officer of Health, Lincoln, whose paper on Welfare Work is summarised in these columns (p. 303).

Some of the figures given by the chairman form striking illustrations of one or two branches alone. Typhus, which for ages had been the scourge of armies, was kept wholly at bay; enteric fever, which ravaged the troops in South Africa— notwithstanding the flooded trenches of Flanders and the great congestion of armies—received a knock-out blow in 1915. The following figures speak for themselves. In South Africa the cases of the typhoid group were 60,000, and deaths 8,000. Our French allies in the first fifteen months of the European war had 96,000 cases, and deaths 12,000; while our records show in France, Egypt, Salonika, Italy and Mesopotamia 15,800 cases and 766 deaths. If the South African standard had been maintained, there would have been 1,200,000 cases during the war, and 160,000 deaths.

The expansion of the corps was gigantic. Consisting in July 1914 of 800 officers and 9,000 other ranks, it developed to 10,000 officers and 132,000 other ranks in 1919, exceeding in numbers the original expeditionary force. Beyond these thousands of busy practitioners attended hospitals for hours daily, refusing all remuneration; 18,000 V.A.D.s gave their services in these hospitals for years together; and 2,000 masseuses, provided by the public spirit of Lord Queenborough, served under the direction of Miss French. With these reinforcements the Army Medical Corps, having 2,000 patients in hospital in 1914, attended 577,000 in 1919, and as early as July 1916 received 48,000 patients in hospital in a single week. For these accomplishments, the Earl of Midleton gave credit to the administrative genius and scientific skill of Sir Alfred Keogh, and to the devoted services of men like Sir John Goodwin and Sir G. Makins to whom military rank could add no eminence.

#### Eminent Civilian Services.

An eloquent tribute from Mr. Churchill followed. Seven thousand beds, he said, were available when war began and there were nearly 700,000 when war ended. That alone measured the expansion in bulk, quantity, and in numbers which the medical science of Great Britain, in military garb, was able to produce. But beyond that, the method of treatment for the great mass of suffering misery and shattered figures that came pouring back on the science, mercy, and humanity of the nation, underwent an advance which well compares with the magnification and multiplication. " It would be wrong," concluded the War Secretary, " for anyone to dwell upon the achievements of the Royal Army Medical Corps without making the fullest acknowledgment of the immense services that were rendered by the eminent civilians who so freely and so generously and so blithely gave their services in this troubled time through which we have passed. British medical science has no doubt gained enormously by the experience of this terrible time, and the R.A.M.C. could not have achieved the great feats either in the

THE PUBLIC WELL-BEING—(*continued*).

domain of medicine or surgery, or in that of military hygiene which are upon record, if they had not received this immense reinforcement from the finest scientific brains in the country devoted to the healing of wounds and suffering."

Sir Alfred Keogh, in reply, passed on most of the credit to others for the long endeavours which had made efficient organisation possible, and he specially associated with the R.A.M.C. and the eminent civilian doctors the great Red Cross Society. Sir John Goodwin and Sir George Makins also returned thanks, their names having been appropriately coupled with the toast proposed.

## Figures and Tables

**Figure f1:**